# Surveys on Exposure to Reptile-Associated Salmonellosis (RAS) in the Piedmont Region—Italy

**DOI:** 10.3390/ani12070906

**Published:** 2022-04-01

**Authors:** Arianna Meletiadis, Cristina Biolatti, Davide Mugetti, Teresa Zaccaria, Raffaella Cipriani, Monica Pitti, Lucia Decastelli, Francesca Cimino, Alessandro Dondo, Cristiana Maurella, Elena Bozzetta, Pier Luigi Acutis

**Affiliations:** 1 Experimental Zooprophylactic Institute for Piedmont, Ligury and Valle d’Aosta, 10154 Turin, Italy; arianna.meletiadis@izsto.it (A.M.); davide.mugetti@izsto.it (D.M.); monica.pitti@izsto.it (M.P.); lucia.decastelli@izsto.it (L.D.); francesca.cimino@izsto.it (F.C.); alessandro.dondo@izsto.it (A.D.); cristiana.maurella@izsto.it (C.M.); elena.bozzetta@izsto.it (E.B.); 2 Azienda Sanitaria Locale CN1, 12100 Cuneo, Italy; cristina.biolatti@gmail.com; 3 Laboratory Diagnostics Department, Microbiology and Virology, University of Turin, 10126 Turin, Italy; tzaccaria@cittadellasalute.to.it (T.Z.); rcipriani@cittadellasalute.to.it (R.C.)

**Keywords:** *Salmonella*, reptiles, RAS, public health, zoonoses

## Abstract

**Simple Summary:**

Reptile-associated salmonellosis (RAS), *Salmonella* infection (salmonellosis) in humans, is acquired through contact both directly with reptiles and indirectly with their environment. In Italy, like in other countries, reptiles have become popular pet animals, but epidemiological data about RAS are not collected. To fill this gap, surveys to estimate the presence and frequency of RAS and human exposure were carried out in Piedmont, a Northern-Italian region. Two studies were conducted among patients with sporadic salmonellosis (i.e., not linked to foodborne outbreaks): the first, restricted to a big city hospital, showed a prevalence of RAS of 7%, and the second, extending to the population affected by sporadic salmonellosis across all the region, showed a prevalence of 3%. In addition, an ocular survey taken in public places displaying reptiles detailed possible exposure through direct and indirect contact and a questionnaire survey that collected data from the general population, including reptile owners, revealed that preventive measures are not always known or applied. These results confirmed that RAS in Italy is present and constitutes a proportion of the human salmonellosis cases in line with the percentages reported in other countries. People should be more informed about RAS and the related preventive measures.

**Abstract:**

Reptile-associated salmonellosis (RAS), *Salmonella* infection in humans, is acquired through contact with reptiles. Reptiles have become popular pet animals, and RAS is likely to be an underestimated but growing problem. No epidemiological data about RAS are routinely collected in Italy. In order to estimate the occurrence of RAS in the Italian human population and to investigate the exposure, two epidemiological studies on patients with sporadic salmonellosis were carried out in the Piedmont region, along with an evaluation of human exposure in public places displaying reptiles and with a survey on people awareness. RAS appeared make up 7% of sporadic salmonellosis in the first study and 3% in the second, more extensive study. A prevalence of 11.7% and 5.7%, respectively, were calculated for the age range of 0–21 years. It was observed that in public places displaying reptiles, it was possible to easily come into contact with the animals and their environment. Some knowledge about RAS emerged from the interviews with the general population, but preventive measures are not completely applied by reptile owners. In conclusion, RAS in Italy is present and constitutes a proportion of the human salmonellosis cases in line with the percentages reported in other countries. Exposure to reptiles should always be considered as a risk factor, and people should be more informed about RAS and the related preventive measures.

## 1. Introduction

*Salmonella* spp. is a Gram-negative bacterium belonging to the *Enterobacteriaceae* family. According to the World Health Organization (WHO), *Salmonella* is one of the major global causes of diarrheal diseases associated either with the consumption of contaminated food products of animal origin or with direct or indirect contact with animals [1]. Reptile-associated salmonellosis (RAS) is called *Salmonella* infection in humans that is acquired through contact with reptiles. In fact, reptiles can normally harbour a wide variety of serovars in their gastrointestinal tract, even simultaneously, such as *S. enterica* serovar Typhimurium, serovars of *S.* Bongori, and *S. enterica* subspecies I, II, IIIa, IIIb, IV, and VI [2,3,4,5]. The bacteria are excreted intermittently through faeces by reptiles. The adaptation to the reptile–host, in which infection occurs asymptomatically and only occasionally causes disease or death, makes the identification of infected animals extremely difficult [2,6]. The infection transmission routes for humans are direct contact with reptiles and/or indirect contact with their environment. Infected people may experience a range of symptoms from diarrhoea to vomit and fever. Children, the elderly, and immunocompromised individuals are at high risk of severe symptoms, even bloodstream infections, that can be life-threatening [1,2]. Some of the strains isolated in reptiles showed resistance to at least one antibiotic, and others were multidrug-resistant strains [7]. Consequently, all reptiles kept as pets must be considered both a potential source of infection for humans and a cause of the increased risk of therapeutic failure in cases of potentially lethal salmonellosis both in human and in veterinary medicine [8,9].

The concern of human exposure risk to RAS is due to the increased and continuous growth of the trade market of reptiles kept as pets. Despite some species being under the Convention on International Trade in Endangered Species (CITES), a large number of species can be easily purchased; thus, during the last 20 years, species of the orders of Squamata and Testudinata have become increasingly popular pet animals [10,11]. In 2005, the European Union was the largest importer of live reptiles, for an approximative import value of seven million euros [12]. In the USA, 4.7 million households own a reptile [2,13]. While in the USA RAS is regularly notified and represent the 6% of sporadic salmonellosis, scant information is available about the European situation, as a uniform notification system is not currently applied [14,15]. To the best of the authors’ knowledge, also for Italy no data are available on the epidemiological situation of RAS. In Italy, in contrast to the USA, there is no ban on the sale of turtles with shell lengths less than 4 inches, a measure aiming at lowering the risk in children [16]. Furthermore, there is no evidence that the Italian population is fully aware of RAS. In the last decade, studies evidenced the presence of *Salmonella* spp. both in wild and captive reptiles in Italy and RAS was usually described as case reports [3,17,18,19,20]. Two of them, conducted by Corrente et al. in 2017 [6] and Sangiorgio et al. in 2016 [21], identified a high prevalence of *Salmonella* among pet reptiles in Southern Italian regions (57% and 46%, respectively) and highlighted some risk factors for owners, linked to the management of the animals. These data are important to underline how salmonellosis associated with reptiles could be a widespread problem in Italy and that it should not be neglected.

Given that, many studies already demonstrated the frequent occurrence of *Salmonella* in animals and in pet reptiles too, the present work aimed to estimate the occurrence of RAS in the human population and to investigate the exposure. To reach these goals, two actions were undertaken: (1) the identification of RAS among human salmonellosis cases and (2) the evaluation of human exposure in public places displaying reptiles and of people’s awareness. For reasons of accessibility to the data and of the feasibility of the on-field studies, the work was carried out in the Piedmont region (North-Western Italy): no biases were identified in this choice, given that no differences were identified in this region compared to the rest of Italy, in terms of specific RAS monitoring and reporting, the application of RAS preventive measures, and the presence of pet reptiles.

## 2. Materials and Methods

### 2.1. Prevalence of RAS among Salmonellosis Cases

Two studies were carried out on two different populations, both covering a 12-month period before the SARS-CoV pandemic.

The first study considered all the sporadic (i.e., not linked to foodborne outbreaks) human salmonellosis cases reported to the Hospital “Città della Salute e della Scienza” of Turin, Italy. A questionnaire aimed at identifying whether direct or indirect contacts with reptiles occurred (Form S1: in Supplementary Material) was administered through a telephone survey to every patient with salmonellosis who agreed to join the study. It was asked whether the infected people: (1) kept pet reptiles or amphibians in the house; (2) often entered places where pet reptiles or amphibians were present; (3) have been, in the days before the onset of the symptoms, in pet shops, zoos or other places where reptiles/amphibians were kept. Personal and anamnestic data, information about symptoms and their onset and exposure to well-known risk factors other than reptiles, were also recorded. In case a pet reptile was present in the house, authorization to collect biological samples by a trained operator was requested, including oral and cloacal swabs of the animal(s), environmental swabs of the case/terrarium/aquarium and of sinks/tubs/basins used for cleaning.

In the second study, all the Regional Food and Nutrition Hygiene Services, SIAN (Piedmont, Italy), who are in charge of epidemiological investigations on foodborne diseases, were recruited, asking them to add, in case of sporadic salmonellosis, a few questions regarding potential contacts with reptiles/amphibians in the five days preceding the onset of symptoms, (Form S2: in Supplementary Material). In the case of positive answers, the first questionnaire (Form S1) was repeated, including the request for authorization to take biological samples.

In both studies, only cases where there was contact with reptiles in the absence of other risk factors were considered as RAS and used to calculate the prevalence of RAS.

When swabs were collected from the animals and their environment, the isolation of *Salmonella* spp. was attempted according to International Organization for Standardization 2017 [22]. The serovars of all isolates were identified using the Kauffmann–White [23] and Le Minor [24] classification scheme for the identification of somatic and flagellar antigens.

Strains serotyped as *S. enterica* ser. Typhimurium/*S. enterica* ser. Typhimurium monophasic variant (4, [5], 12:i:-) were tested with multiplex PCR, according to Lim et al. [25], to determine the presence of four genes: rfbJ, fliB, invA and fliC. A field strain of *S. enterica* ser. Typhimurium DT 104, positive for rfbJ, fljB invA and fliC genes, and one of *S. enterica* ser. Typhimurium (4, [5], 12: i:-), positive for rfbJ, invA and fliC, both supplied by the National Reference Center for Salmonellosis (Padua, Italy), were used as positive controls.

When possible, Pulsed-field Gel Electrophoresis (PFGE) was carried out to compare the isolate from a patient with that from his pet reptile. The PFGE protocol adopted by PULSENET was applied [26]. The obtained PFGE patterns were compared using Bionumerics version 7.6 software [27], with the Dice coefficient [28] with 2% band tolerance and 2% optimization and the unweighted pair group method with arithmetic averages (UPGMA) [29]. *Salmonella* strains with 100% similarity were considered identical and were assigned to the same PFGE type.

### 2.2. Evaluation of Exposure to RAS and of People’s Awareness

#### 2.2.1. Evaluation of the Human Exposure in Public Places Displaying Reptiles

To evaluate the exposure to *Salmonella*, a survey was carried out across the region for public places and events displaying reptiles, mainly pet shops and exhibitions. An observer visited these places and recorded (Form S3: in Supplementary Material) data about the location (number of hosted reptiles; the presence of physical barriers between animals and visitors; the presence of signposting or other types of instructions warning about risks; the presence of disinfectant dispensers) and data concerning the occurrence of direct or indirect contacts between customers/sellers and the animals. It was observed how many times people touched reptiles or potentially contaminated objects and their subsequent behaviour (e.g., touching eyes, nose, mouth, body, clothes, other persons, other animals, smartphone, money) along with eventual hand washing or disinfection.

#### 2.2.2. Evaluation of People’s Awareness about RAS

A questionnaire (Form S4: in Supplementary Material) was designed to interview adults residing in the region to assess the level of awareness about the risk of contracting RAS. The survey was open for 7 months, and it was advertised on the website of the Experimental Zooprophylactic Institute for Piedmont, Ligury and Valle d’Aosta, on social media and during public events, organized to communicate science to the general population. Interviewees participated voluntarily. Different questions were asked depending on whether the interviewee was a reptile owner or not. In the first case, questions regarded the number and the species of owned animals, hygiene practices, how animals were managed in terms of feeding, the possibility of wandering in the house, the location of the terraria/aquaterraria and cleaning procedures. If they were not a reptile owner, they were asked whether they intended to buy one and, if so, which species. All interviewees were asked whether they owned any other species of animals. Finally, they were asked whether they knew about salmonellosis, the related risk factors and its possible transmission from reptiles.

#### 2.2.3. Statistical Analysis

Data were collected in an ad hoc database and analyzed with statistical software, Stata 16.1 [30]; the prevalence and the binomial confidence interval were calculated as the number of positives out of the tested population who accepted to be included in the study.

## 3. Results

### 3.1. Prevalence of RAS among Salmonellosis Cases

**Figure 1:**
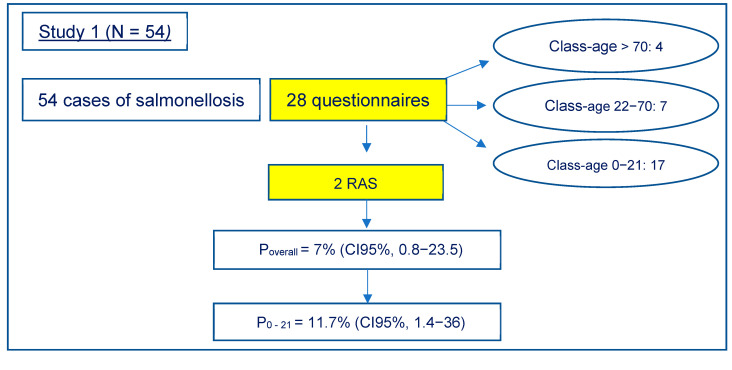
Results of the study carried out at the Hospital “Città della Salute e della Scienza” of Turin, Italy.

In the first study, *Salmonella* was isolated from the stool samples of 54 symptomatic patients, and 28 were accepted to be included in the epidemiological survey (Figure 1). The age of these 28 patients ranged from 6 months to 83 years: 61% (17/28) were between 0−21 years, 25% (7/28) between 22−70 years and 14% (4/28) > 70 years.

Twenty-seven samples out of twenty-eight were typed; the most represented was *S. enterica* ser. The Typhimurium monophasic variant (4, [5], 12:i:-) had nine isolates; the others were *S. enterica* serovars Winston, Typhimurium, London, Napoli, Pakistan, Enteritidis, Infantis, Give, Dabou, Newport, Derby and Kentucky.

Most of the 28 subjects were exposed to other risk factors such as having eaten raw food (meat, eggs, milk, shellfish); having had contact with animals other than reptiles or amphibians.

In four cases, contact (direct or indirect) with reptiles during the 5 days before the onset of the symptoms was reported: a child, 7 years old, played with turtles along a pond and resulted in being infected with *S. enterica* ser. Typhimurium monophasic variant (4, [5], 12:i:-); a 6 month old infant infected with *S. enterica* ser. Kentucky, brought by his parents to a house hosting freshwater turtles; a 74-year-old man, infected with *S. enterica* ser. London, who owned a freshwater turtle; a 2-year-old girl, infected with *S. enterica* ser. Typhimurium monophasic variant (4, [5], 12:i:-), who visited zoos/pet shops where reptiles were present. No biological samples could be collected for animals potentially related to these cases. The 74-year-old man and the girl ingested possibly at-risk food during the three days before the start of the symptoms, unlike the two children for whom no risk factors other than the contact with reptiles were identified. Therefore, only two cases were considered as RAS.

**Figure 2:**
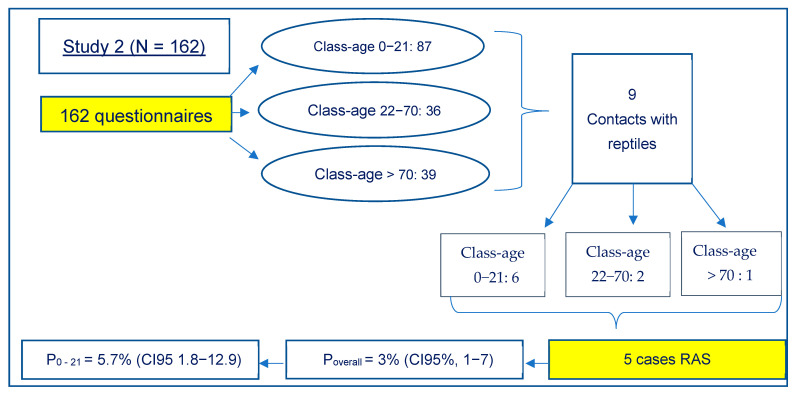
Results of the study carried out at the regional level.

In the second study, the involved SIAN returned 162 complete questionnaires (Figure 2).

From these results, it appeared that 87 subjects affected by *Salmonella* spp. were aged between 0 and 21 years, 36 between 22 and 70 years and 39 over 71 years. Contact with reptiles was reported in nine cases. Six of them were aged between 0 and 21 years, 2 between 22 and 70 years and one over 71 years. In eight of these cases, the marsh turtle Trachemys scripta was involved, while in one case, an episode of contact with wild tortoises was reported.

Four cases were subsequently excluded.

In detail:

- Case 1: the patient did not agree to participate in the more extensive questionnaire;
- Case 2: the animal’s swabs resulted negative, and the patient was exposed to other risk factors;
- Case 3: the patient was exposed to other risk factors;
- Case 4: the samples both from the animal and the patient tested positive during analysis, but PFGE showed that different strains were involved (*S. enterica* ser. Lome for the animal and another not identified variant for the boy).

Five cases could be considered RAS instead, and, in four of them, it was possible to collect samples from the animals and their environment in the households.

In detail:

- Case 1: a 14-year-old child had contact with a marsh turtle hosted at home. The aquarium was washed and cleaned directly in the bathtub. *S. enterica* ser. Typhimurium monophasic variant (4, [5], 12:i:-) was isolated from environmental swabs, and PFGE confirmed the identity of the strain isolated in the patient;
- Case 2: a 2-year-old child had contact with a marsh turtle hosted at home. The family used to throw the water and clean the aquaterrarium in the sink of the kitchen. No other risk factors were present. *S. enterica* ser. Pomona was isolated from swabs obtained from the animal and the water of the aquaterrarium. The isolate from the patient could not be retrieved for further investigation, and the serovar was not determined at the hospital where the diagnosis was made;
- Case 3 and 4: a 7-year-old boy and a 10-year-old girl, both had contact with pet marsh turtles. The aquariums were cleaned in the garden and the kitchen sink, respectively. All samplings were negative, but cases were recognized as RAS considering the possible intermittent elimination of Salmonella by reptiles and the absence of other risk factors;
- Case 5: a 16-year-old boy had contact with a marsh turtle hosted at home, but he did not agree to answer the more extensive questionnaire. The SIAN doctor declared the absence of other risk factors; therefore, the episode was considered RAS.

To summarize, in the first study, RAS appeared to have a prevalence P = 7% (CI95%, 0.8−23.5) of sporadic salmonellosis, while in the second study, the prevalence was 3% (CI95%, 1−7). Looking at the age-specific prevalence, we calculated a P = 11.7% (CI95%, 1.4−36) and 5.7%, (CI95%, 1.8−12.9), respectively, for the age range of 0−21 years.

### 3.2. Evaluation of Exposure to RAS and of People’s Awareness

#### 3.2.1. Evaluation of the Human Exposure in Public Places Displaying Reptiles

A total of 460 min of observation was collected in three pet shops, three exhibitions and one animal show keeping reptiles. None of these places had signposting or another type of information warning about possible risks and recommending the adoption of precautional hygiene measures. In five places, no barriers were present to prevent visitors from direct contact with animals. In total, 77 contacts were recorded during the observation period, 86% of them made by visitors and 14% by sellers. In total, 90% were indirect contacts and mostly involved the vivarium (88% of the indirect contacts). After the indirect contact, people, without hand washing, touched: body/clothes (49%), other people (14%), other things (14%), mouth/nose/eyes (9%), other head points (6%), smartphones (5%) and money (1%).

#### 3.2.2. Evaluation of People’s Awareness about RAS

In total, 134 questionnaires were completed: interviewees were 73% females and 25% males (2% did not specify sex). The mean age was 43 (17–72). Thirteen of them owned reptiles at the time of the questionnaire: six owned a freshwater turtle (genus Trachemys or Graptemys), three a tortoise, the other three an iguana, a pogona and a Japanese newt, respectively. One interviewee owned both a snake and a frog. On average, the animals had been housed for 9.5 years. In 69% of these 13 cases, reptiles/amphibians were kept inside the house: 7% had access to the kitchen, while 15% had access to the bathroom. Thirty-one percent of the owners temporarily placed their pet in the kitchen or bathroom sink, bathtub or shower. The cleaning of the aqua/terrariums took place with variable frequency, from occasionally to every 2/3 days and was carried out mainly in the bathroom (38%) or outside (23%). Only 31% of owners used disposable gloves for cleaning, but 69% said they sanitized the place where the aqua/terrarium and other items related to the reptile/amphibian were cleaned. Sixty-nine percent of interviewees always washed their hands after touching their pet, 23% not always, while 8% admitted to never washing them. No owner reported bringing the reptile/amphibian close to the face or mouth. Regarding pet food, 46% of the owners fed their pet (turtles or iguanas) with fresh vegetables prepared in the kitchen; 23% (50% of the turtle owners) purchased shrimps sold as turtle food; 15% fed their pet dried or frozen insects prepared in kitchen; 7.6% (all the snake owners) used live crickets or feeder mice; 15% used industrial pet food.

Of the 121 interviewees who did not own reptiles/amphibians at the time of the questionnaire, 6 would have liked to acquire one, and the desired animals were turtles, bearded dragons, iguanas, chameleons, geckos and boa snakes.

In total, 45.5% of all the interviewees owned other animals at the time of the questionnaire: 25% cats, 18% dogs, 4% aquarium fish, 5% rodents and 1% psittacines. Approximately, 10% owned pets of different species. Considering only the owners of reptiles/amphibians, 38% also hosted animals of other species (dogs, cats, fish and psittacines).

Overall, 69% of all interviewees washed their hands after touching any animal, while 76% washed their hands after touching animal-related objects. In total, 95.5% of interviewees knew salmonellosis as a disease. The most known carriers of *Salmonella*, other than reptiles, were eggs (78%), raw or undercooked meat (27%), fish (9%), milk and derivatives (8%), unwashed vegetables (7%), shellfish (3%) and contaminated water (3%). Reptiles were recognized as a possible source of salmonellosis by 70% of all the interviewees and 84.6% of all the reptile owners.

## 4. Discussion

According to the EFSA report 2021 [31], confirmed cases of salmonellosis in humans in 2019 were 87,923, with an EU notification rate of 20.0 cases per 100,000 inhabitants and a fatality rate of 0.22%. In Italy, 3268 cases were recorded. No data are available on what percentage of RAS is included in these numbers, and it does not appear that systematic epidemiological investigations and reporting are in place in most of the European countries, Italy included. In 2008, the Editorial team Collective of Eurosurveillance tried to give a picture of the situation of RAS in Europe, circulating a quick survey among their journal’s editorial advisors to collect data on the occurrence of such cases in European countries. In this paper, which seems to be the most comprehensive available in scientific literature, no prevalence data are present, and only isolated cases were reported in the different countries, identified through passive surveillance. Some countries said they never knew about cases of RAS in their territory. Italy was not mentioned. The authors concluded that the presented data were far from being complete and uniform and that although known cases attributed to exposure to reptiles and other exotic pets may constitute a small proportion of all human cases of salmonellosis, it is likely to be an underestimated but growing problem that merits more attention [14].

The present study was thus carried out starting from this premises. In the epidemiological investigation conducted in collaboration with the Laboratory of Microbiology and Virology of the City of Health and Science of Turin, 4 out of 28 patients affected by salmonellosis had contact with reptiles in the five days preceding the onset of symptoms.

According to the adopted criteria of classification (reptiles/amphibians as the only reported risk factor), only two cases were considered RAS, showing a point prevalence in the studied population of 7%, rising to 11.7% if only the under 21 age group is considered, similarly to what was reported in the United States (11% in subjects under the age of 21) [32]. When the study was extended to the Piedmont area, with the collaboration of SIAN, the percentage of RAS decreased to 3% of the total sporadic salmonellosis cases and 5.7% considering subjects under the age of 21. To the authors’ knowledge, this is the first estimation of the prevalence of RAS in Italy on the total of the sporadic salmonellosis cases. There are no reasons to think that these percentages, obtained on a regional basis, cannot be extended to the national situation. It is, instead, probable that even in the present study, although based on epidemiological criteria, prevalence is underestimated, as some subjects were excluded because they had other concomitant risk factors. Furthermore, one case was excluded because PFGE showed differences between isolates of the patient and his pet. However, given that reptiles can harbour multiple serotypes, it is possible that at the time of sampling, the animal was shedding a serotype different from that transmitted to the child [2,3,6].

In all the seven RAS cases described in the present study, freshwater turtles were identified as the possible source of infection. However, from the data obtained from the questionnaire used to evaluate awareness about RAS, it appeared that nearly half of participating reptile owners harboured marsh turtles, while others owned tortoises, amphibians, saurians and snakes. Indeed, lizards and snakes are increasingly desired by reptile lovers. Consequently, as demonstrated in the review by Whiley et al. [33], salmonellosis associated with those reptiles can be an emerging global issue of public health concern that can also cause a shift towards adulthood in the age group at risk.

One of the main risks for RAS is the lack of the general public’s knowledge regarding hazards associated with reptiles [33]. In Italy, pet store owners or exhibitors of reptiles are not required to provide information regarding the risk of RAS to the public. From the observations collected in the present study, it was clear that, in public places displaying reptiles, it is possible to come into contact not only with potentially contaminated surfaces but also with the animals themselves. Barriers were absent or insufficient to prevent contacts, no hygiene rules were prescribed, and, in 460 min of observation, approximately one contact every six minutes was recorded, and in none of those cases did the subject clean their hands after touching the possibly contaminated surface or the animals. These data showed that the risk of exposure to RAS is uncontrolled in such settings.

From the distributed questionnaires, it emerged that 10% of the interviewees owned a reptile, a much higher percentage than the 4% recorded in the 1970s in the United States, when the public struggle against the RAS officially began and a ban on the sale of turtles with a carapace length of less than 4 inches was introduced [34]. The reptile owners appeared to be quite careful regarding some hygiene practices recommended for the prevention of RAS: no one reported bringing the animal close to their face or their mouth, while most of them washed their hands after touching their pet and sanitized the place where the display case and other objects of the animal were cleaned (69% in both cases). This group of owners seems more aware than the sample interviewed by Sangiorgio et al. [21] in Puglia and Basilicata and Corrente et al. [6] in Puglia, in which 26% and 12%, respectively, of individuals, admitted they put their face in contact with the animal and 44% and 65%, respectively, had the habit of always washing their hands. However, less care on other aspects emerged that are in line with the other studies: only 31% of the interviewees use disposable gloves for cleaning (20% in the study of Sangiorgio et al. and 21% in the study of Corrente et al.) and the same number also temporarily put their reptile/amphibian in sanitary ware used for personal hygiene or food preparation (24% Sangiorgio et al., and 26% Corrente et al.). Regarding data about feeding practices, it must be noted that, in this study, all the snake owners used feeder mice, in contrast with the study of Corrente et al., where 48% of interviewees regularly fed their reptiles with live rats or mice. Feeder mice themselves can be a risk factor for human salmonellosis: from 2012 to 2016, 278 salmonellosis human cases were identified, in England and Denmark, associated with *S. enterica* ser. Enteritidis PT8 outbreaks due to feeder mice [35].

However, Salmonellosis is well known by 95.5% of all the interviewees (38% in the samples of Sangiorgio et al., and 55% by Corrente et al.), and it appeared that a large proportion of them, higher in the group of reptile owners, knew that reptiles can be a source of infection.

In conclusion, our study showed that RAS in Italy are present and constitute a proportion of the human salmonellosis cases in line with the percentages reported in other countries [5,14]. No measures to mitigate the risk of RAS is in place in Italy, given the absence of import restrictions, the lack of systematic information for reptile owners and prescriptive hygiene measures, which have also emerged from the observational study. A key factor for RAS prevention is the information of the general public and the sensitization of physicians and public health experts [14]. A decrease in RAS cases due to a national information campaign was well documented in Sweden [36]. To try to fill this gap in Italy, an educational campaign, designed to attract mainly children, was implemented by the the authors, through the development of a website in which RAS is explained, and guidelines of good hygiene and management practices for RAS prevention are promoted (http://www.scuolachannel.it/projects/home/unrettileperamico/).

## Data Availability

Not applicable.

## Supplementary Materials

The following supporting information can be downloaded at:https://www.mdpi.com/article/10.3390/ani12070906/s1, Form S1: questionnaire administered to patients with salmonellosis, aimed at identifying whether direct or indirect contacts with reptiles occurred; Form S2: questionnaire administered to patients with sporadic salmonellosis, regarding potential contacts with reptiles/amphibians; Form S3: record sheet of the survey carried out in public places displaying reptiles; Form S4: questionnaire administered to people for the evaluation of people’s awareness about RAS.
