# Supplementary material for: Surveys on Exposure to Reptile-Associated Salmonellosis (RAS) in the Piedmont Region—Italy
Source: Animals (Basel). 2022 Apr 1;12(7):906. doi: 10.3390/ani12070906 (PMC8996925; doi:10.3390/ani12070906)
Supplement: Supplementary file 1 [file animals-12-00906-s001.zip › animals-1622382-supplementary.pdf]

FORM 1: questionnaire administered to patients with salmonellosis, aimed at identifying whether direct or indirect contacts with reptiles occurred.

Questionnaire n° |__|__| year |__|__|__|__| Date |__|__|/|__|__|/|__|__|

Strain identification code ____

Start time of the interview |__|__|:|__|__|

SYMPTOMATOLOGY

Fever	YES|__| NO|__|	Diarrhoea	YES|__| NO|__|
Nausea	YES|__| NO|__|	Headaches	YES|__| NO|__|
Vomit	YES|__| NO|__|	Abdominal Cramps	YES|__| NO|__|

COURSE OF THE DISEASE

Date of symptoms onset |__|__|/|__|__|/|__|__| Duration of symptoms (in days) |__|__|

Was hospitalization necessary? YES|__| NO|__|

Did other members of the family present the same symptoms, even mild? YES|__| NO|__|

RAS RISK FACTORS

Are reptiles present in the house? YES|__| NO|__|

Are amphibians present in the house? YES|__| NO|__|

If YES

- How many? |__|__| What species? ____________________________
  ____
  ____
- How old are the animals? ____
- From how long are they present in the house? ____________________________
- Where is the terraria/acqua-terraria placed? ____________________________
- Do the animals have access to the kitchen? YES|__| NO|__|
- Do the animals have access to the bathroom? YES|__| NO|__|
- Are the animals temporarily left in the bathtub or in the kitchen? YES|__| NO|__|
- What type of petfood are they fed with? ________________________________
- Is it fresh or frozen? ____
- Where is the petfood prepared? ______________________________________
- How often are food and water containers cleaned? __________________________

- Where are food and water containers cleaned? ______________________________________
- How often is the terrarium / aquarium cleaned? _____________________________________
- Where is the terrarium / aquarium cleaned? ____
- Does the person in charge of cleaning use disposable gloves? YES|__| NO|__|
- Is the place, where the objects relating to animals are cleaned, sanitized after use?_____
- Do you agree to be called by the veterinarians of the Istituto Zooprofilattico Sperimentale of Turin for the eventual collection of biological samples from the animals (e.g. faeces, aquarium water, environmental swabs, cloacal swabs)? YES|__| NO|__|

Did you have contact with reptiles (including wild ones) in the 5 days prior to the onset of symptoms? YES|__| NO|__|

Did you have contact with amphibians (including wild ones) in the 5 days prior to the onset of symptoms? YES|__| NO|__|

If YES

- What species was involved? ____

  ____

  ____
- Where did you come in contact ? ____
- Did you touch the animal with your hands? YES|__| NO|__|
- Did you kiss the animal or bring it close to your face? YES|__| NO|__|

Have you been guest in homes / places where there are reptiles? YES|__| NO|__|

Have you been in contact with people who have reptiles (family members, friends, baby sitters, domestic workers)? YES|__| NO|__|

Have you been in pet shops? YES|__| NO|__|

Have you been in zoos or other animal exhibitions / fair YES|__| NO|__|

Did you have contacts at home or elsewhere with animals other than reptiles / amphibians?

YES|__| NO|__|

If yes, did you wash your hands after touching any animal? YES|__| NO|__|

Did you wash your hands after touching objects related to an animal (e.g. bowls, litter boxes, kennels, leashes, etc.)? YES|__| NO|__|

In the 3 days prior to the start of symptoms, did you eat raw eggs or foods containing raw eggs (e.g. tiramisu, mayonnaise or other creams) YES|__| NO|__|

In the 3 days prior the start of symptoms, did you eat other foods of animal origin such as:

- raw or undercooked meat YES|__| NO|__|
- cured meat YES|__| NO|__|
- raw milk or cheese made from raw milk YES|__| NO|__|
- raw shellfish YES|__| NO|__|

GENERAL PATIENT DATA

Date of birth |__|__||__|__||__|__| Sex M |__| F |__|

Number of family members |__|__|

District of residence |__|__|

Time of interview |__|__|:|__|__|

FORM 2: questionnaire administered to patients with sporadic salmonellosis, regarding potential contacts with reptiles/amphibians.

The questionnaire must be sent for all cases of confirmed salmonellosis (even for cases in which the answer NO to the first question is given)

SIAN carrying out the survey _____

Case of confirmed salmonellosis with the serotypes __________________________ date __________

Sex ☐M ☐F

Age___________

1) Do you host reptiles/amphibians at home or have you been in places where reptiles/amphibians are present (both captive and wild) in the five days prior to the onset of symptoms?	☐YES<br>☐NO
If ''YES''	
2) Where?	☐Home<br><br>☐Other place. Describe it_____________
3) What species were involved?	___________________
4) Did you touch the animal?	☐YES<br>☐NO
5) Do you agree to be called by veterinarians of Istituto Zooprofilattico Sperimentale of Turin for an interview?	☐YES<br>☐NO
In case you agree, please insert name and contact details (telephone; e-mail)	

Form 3: record sheet of the survey carried out in public places displaying reptiles.

PLACE: DATE|__|__|/|__|__|/|__|__|
OBSERVER:

SETTING:
Are there any signs indicating not to touch the animals or their enclosures? YES|__| NO|__|
Are there any signs recommending to wash hands after touching the animals or their enclosures? YES|__| NO|__|
Are hand sanitizer dispensers available? YES|__| NO|__|
Is there any information about the risks of RAS? YES|__| NO|__|
Is there any information about precautionary hygiene practices? YES|__| NO|__|
Are there physical barriers that isolate animals from accidental contacts? YES|__| NO|__|
Are the animal groups housed in group in restricted places? YES|__| NO|__|

STARTING TIME of the observation: |__|__|:|__|__|
ENDING TIME of the observation: |__|__|:|__|__|

Number of the observation	1	2	3	4	5	6	7	8
Seller								
Visitor								
Direct contact								
Indirect contact (specify the object)								
Indicate if the person touches the eyes, mouth, nose								
Indicate if the person touches the body, clothes, bag								
Indicate if the person touches another person								
Indicate if the person touches another animal								
Indicate if the person cleaned his hand								

FORM 4: questionnaire administered to people for the evaluation of people's awareness about RAS.

Are you a reptiles/amphibians owner?

1) If YES:

- How many reptiles/amphibians are present in your home?
- What species?
- How old are the animals?
- From how long they are present in your house?
- Do you regularly wash your hand after touching the animal?
- Do you regularly wash your hands after touching any object related to the animal (bowls, toys, terrarium, etc..)?
- Is it possible that your face comes in contact with the animal when you manipulate it?
- Where is the terrarium/aquarium placed in the house?
- Do the animals have access to the kitchen?
- Do the animals have access to the bathroom?
- Is it possible that the animals are left, even temporarily, in the bathtub or the kitchen sink?
- What type of food are they fed with?
- Are the ingredients fresh or frozen?
- Where is the pet food prepared?
- How often are the bowls cleaned?
- Where are the bowls cleaned?
- How often is the terrarium/aquarium cleaned?
- Does the person in charge of cleaning use disposable gloves?
- Do you sanitize surfaces on which you cleaned the objects related to the animal (bowls, toys, terrarium, etc..)?
- Do you have animals of other species?

  If YES:
  - what species?
  - do you regularly wash your hands after touching any object related to the animal (bowls, toys, etc..)?
- Do you know what salmonellosis is?
- Do you know what type of food is at more risk of Salmonella transmission?
- Do you know that Salmonella can be transmitted by reptiles?

2) If NO:

- Would you like to have a reptile as a pet?
- If YES, what species?
- Do you have animals of other species?

  If YES:
  - what species?
  - do you regularly wash your hands after touching any object related to the animal (bowls, toys, etc..)?
- Do you know what salmonellosis is?
- Do you know what type of food is at more risk of Salmonella transmission?
- Do you know that Salmonella can be transmitted by reptiles?

Age:
date:
